# Supplementary material for: A simple and economic protocol for efficient in vitro fertilization using cryopreserved mouse sperm
Source: PLoS One. 2021 Oct 28;16(10):e0259202. doi: 10.1371/journal.pone.0259202 (PMC8553151; doi:10.1371/journal.pone.0259202)
Supplement: S1 File — (PDF) [file pone.0259202.s001.pdf]

A simple and economic protocol for efficient *in vitro* fertilization using cryopreserved mouse sperm V.2

Version 2

Magdalena Wigger[1,2], Simon E Tröder[1,2], Branko Zevnik[1,2]

[1]Cluster of Excellence Cellular Stress Responses in Aging-associated Diseases (CECAD), Faculty of Medicine and University Hospital Cologne, University of Cologne, Cologne, Germany;

[2]in vivo Research Facility, Faculty of Medicine and University Hospital Cologne, University of Cologne, Cologne, Germany

dx.doi.org/10.17504/protocols.io.bx2spqee

Magdalena Wigger

ABSTRACT

The advent of genome editing tools like CRISPR/Cas has substantially increased the number of genetically engineered mouse models in recent years. In support of refinement and reduction, sperm cryopreservation is advantageous compared to embryo freezing for archiving and distribution of such mouse models. The *in vitro* fertilization using cryopreserved sperm from the most widely used C57BL/6 strain has become highly efficient in recent years due to several improvements of the procedure. However, purchase of the necessary media for routine application of the current protocol poses a constant burden on budgetary constraints. In-house media preparation, instead, is complex and requires quality control of each batch. Here, we describe a cost-effective and easily adaptable approach for *in vitro* fertilization using cryopreserved C57BL/6 sperm. This is mainly achieved by modification of an affordable commercial fertilization medium and a step-by-step description of all other necessary reagents. This protocol is compatible with frozen sperm from all major repositories and the IVF can easily be adapted to accommodate freshly harvested sperm.

ATTACHMENTS

Protocol scheme.png

DOI

dx.doi.org/10.17504/protocols.io.bx2spqee

PROTOCOL CITATION

Magdalena Wigger, Simon E Tröder, Branko Zevnik . A simple and economic protocol for efficient in vitro fertilization using cryopreserved mouse sperm. **protocols.io**
https://dx.doi.org/10.17504/protocols.io.bx2spqee
Version created by Magdalena Wigger

KEYWORDS

Sperm, Cryopreservation, Embryos, Genetics, Genome editing, In vitro fertilization, Mouse model

LICENSE

This is an open access protocol distributed under the terms of the Creative Commons Attribution License, which permits unrestricted use, distribution, and reproduction in any medium, provided the original author and source are credited

CREATED

Sep 07, 2021

LAST MODIFIED

Sep 09, 2021

PROTOCOL INTEGER ID

53042

GUIDELINES

Citation: Magdalena Wigger, Simon E TrÃÂ¶der, Branko Zevnik ( ). A simple and economic protocol for efficient in vitro fertilization using cryopreserved mouse sperm. https://dx.doi.org/10.17504/protocols.io.bx2spqee

ANIMAL PROCEDURES

Sperm of 10- to 20-week-old C57BL/6 males and oocytes of superovulated 3-4-week-old (i.e. 12-14 g body weight) of the corresponding genetic background are collected for the IVF procedures as decribed in published protocols (Behringer, R., *et al.* Manipulating the mouse embryo: a laboratory manual, Fourth edition. ed. Cold Spring Harbor, New York: Cold Spring Harbor Laboratory Press; 2014.). Females are superovulated with 5 IU of PMSG (Aviva Systems Biology; OPPA01037) followed after 48 hours by 5 IU of hCG (MSD Animal Health; Ovogest 300I.E.).

We routinely pool the sperm from 2 males to compensate for variability in sperm quality between males, generate sufficient quantities of straws for archiving and distribution and enable the optional quality control via an IVF of a single straw for each cryopreservation. Use of a single male is possible as well but the number of straws and volume of media used needs to be reduced by 50%. The integrity of each sperm sample after cryopreservation is recommended to be evaluated by a validation IVF with oocytes from 3 superovulated females. Samples with fertilization rates of >20% can be considered as successfully archived.

The 2-cell embryo transfer is performed unilaterally according to a published protocol (Behringer, R., *et al.* Manipulating the mouse embryo: a laboratory manual, Fourth edition. ed. Cold Spring Harbor, New York: Cold Spring Harbor Laboratory Press; 2014.). Ketamine (100 mg/kg body weight; BW), Ketaset, Zoetis Deutschland GmbH) and xylazine chloride (10 mg/kg BW), XYLAZIN, Serumwerk Bernburg AG) are used as anesthetics and carprofen (5 mg/kg BW) Carprosol, CP-Pharma Handels-Gesellschaft mBH) as analgesic after surgery. Well tolerated inhalational anesthetics like isoflurane may be used instead of ketamine/xylazine chloride.

MEDIA PREPARATION

Use only embryo-grade reagents.
Use weighing paper and disposable spatulas only.
For precise volume measurement during c-TYH preparation use a 25 ml volumetric flask (Vitlab; 671941) washed with embryo-grade water (Sigma; W1503) before first use and between subsequent steps. Discard the flask after the entire procedure. Do not use detergent to clean the flask in order to re-use it as residual detergent will harm the sperm.

gCPA for sperm cryopreservation

Prepare cryoprotective agent (gCPA) containing 18% raffinose pentahydrate and 3% skim milk supplemented with 100 mM L-glutamine as follows:

- Place 0.146 g of L-glutamine in 10 ml of prewarmed (60°C) embryo-grade water and vortex for 3 min
- Add 1.8 g of raffinose pentahydrate and 0.3 g of skim milk, vortex the solution for 3 min and incubate for 90 min at 60°C. Vortex gCPA every 30 min for 3 min
- Centrifuge the solution at 10,000 g for 60 min (e.g. by using 1 ml aliquots) and filter the supernatant through a 0.22 µm filter
- Check osmolality (500-520 mOsm/kg) and store aliquots at room temperature for up to 3 months

c-TYH medium for sperm preincubation

For sperm preincubation prepare c-TYH containing 1.0 mg/ml of polyvinyl alcohol and 0.75 mM methyl-β-cyclodextrin (MBCD) from concentrated stocks as follows (alternatively commercially available FERTIUP® PM (Cosmo Bio; KYD-002-EX-X5) can be used):

1. Prepare a mixture of the following substances for concentrated stock solutions:

- Stock X:
  - 10x NaCl: Weigh 1.744 g of NaCl into a 50 ml centrifuge tube and dissolve in 20 ml of embryo-grade water
  - 100x Salt Mix: Weigh all the reagents into a 50 ml centrifuge tube and dissolve in 20 ml of embryo-grade water

Citation: Magdalena Wigger, Simon E TrÃÂ¶der, Branko Zevnik ( ). A simple and economic protocol for efficient in vitro fertilization using cryopreserved mouse sperm. https://dx.doi.org/10.17504/protocols.io.bx2spqee

A	B
**Reagent**	**Amount**
KCl	0.890 g
MgSO4 x 7 H2O	0.733 g
KH2PO4	0.405 g
D-(+)-Glucose	2.500 g
Penicillin G	0.188 g
Streptomycin	0.125 g

- Rinse a new 25 ml volumetric flask with embryo-grade water before use by inverting the plugged flask
- Transfer the dissolved salt mix solution into the flask, wash the centrifuge tube with 3 ml of embryo-grade water and add it to the flask
- Fill the flask up to 25 ml with embryo-grade water
- Transfer the salt mix solution into a new centrifuge tube
- Pipette 2.5 ml of the salt mix to the centrifuge tube containing 20 ml of NaCl solution
- Wash the previously used 25 ml volumetric flask 3x with embryo-grade water
- Transfer the prepared NaCl + salt mix solution into the flask and fill it up to 25 ml with embryo-grade water
- Filter the solution through a 0.22 µm filter and store 3 ml aliquots at -20°C for a maximum of 12 months

▪ Stock Y: 100x $CaCl_2$ x $2H_2O$

- Weigh 0.628 g of $CaCl_2$ x $2H_2O$ into a 50 ml centrifuge tube and dissolve in 20 ml of embryo-grade water
- Wash the previously used 25 ml volumetric flask 3x with embryo-grade water
- Transfer the $CaCl_2$ solution into the flask, wash the centrifuge tube with 3 ml of embryo-grade water and add it to the flask
- Fill the flask up to 25 ml with embryo-grade water
- Filter the solution through a 0.22 µm filter, prepare 300 µl aliquots and store at -20°C for a maximum of 12 months

2. Preparation of PVA solution:

▪ Fill a 15 ml centrifuge tube with 2.5 ml of embryo-grade water
▪ Weigh 0.025 g of PVA and add it into the centrifuge tube
▪ To dissolve the PVA, heat the solution up to 90°C for at least 10 min and vortex

3. Preparation of c-TYH medium (in 50 ml centrifuge tube)

▪ Pipette 10 ml of embryo-grade water to a new 50 ml centrifuge tube and add to it in the following order:

A	B	C
**Reagent/Stock**	**Amount**	**Comment**
Stock X	2.5 ml	
7.5% NaHCO3, Sodium bicarbonate solution	702 µl	**open sterile**
100mM Sodium pyruvate solution	125 µl	**open sterile**
MBCD	0.0246 g	weigh directly into the solution
PVA solution	2.5 ml	wash the centrifuge tube with 3 ml of embryo-grade water and add it to the prepared c-TYH
Stock Y	250 µl	

▪ Transfer the medium into a 25 ml volumetric flask, wash the centrifuge tube with 3ml of embryo-grade water and add it to the flask
▪ Fill the flask up to 25 ml with embryo-grade water (**CAUTION:** As soon as the PVA solution is added, it starts to foam)
▪ Filter the solution through a 0.22 µm filter and store 1 ml aliquots at +4°C for up to 3 months
▪ Perform osmolality check (283-293mOsm/kg)

mRVF medium for *in vitro* fertilization

For the IVF procedure supplement commercially available Research Vitro Fert (RVF) medium (Cook Medical; K-RVFE-50) with reduced glutathione (GSH; final concentration: 1 mM) and Ca^{2+} (final concentration: 5.14 mM) as following to obtain modified RVF (mRVF) medium:

Citation: Magdalena Wigger, Simon E TrÃÂ¶der, Branko Zevnik ( ). A simple and economic protocol for efficient in vitro fertilization using cryopreserved mouse sperm. https://dx.doi.org/10.17504/protocols.io.bx2spqee

- Prepare a 100x $CaCl_2$ x $2H_2O$ stock solution (310 mM) by dissolving 0.4558 g of $CaCl_2$ x $2H_2O$ in 10 ml of embryo-grade water
- Filter the solution through a 0.22 µm filter and store aliquots at -20°C for a maximum of 6 months
- On the day of IVF thaw an aliquot of $CaCl_2$ at room temperature
- Add 150 µl of 100x $CaCl_2$ to 15 ml of RVF medium and mix gently
- Place 1 ml of RVF medium supplemented with $CaCl_2$ in a tube containing 30.7 mg of GSH and vortex
- Add 50 µl of this solution to 5 ml of RVF medium supplemented with $CaCl_2$, mix gently and filter using 0.22 µm syringe end filter in order to obtain mRVF medium

Note: HTF medium (e.g. Merck MR-070-D) may likely be used instead of RVF and supplemented as described above.

Embryo culture media

Use **M2** medium for handling of embryos outside and **M16** or **KSOM** medium for culture inside a CO_2 incubator.
Many labs prefer the use of KSOM as it supports the development of embryos from many different mouse strains.
All media can be prepared in-house according to the previously published method (Behringer, R., *et al.* Manipulating the mouse embryo: a laboratory manual, Fourth edition. ed. Cold Spring Harbor, New York: Cold Spring Harbor Laboratory Press; 2014.) or commercially purchased.
Mouse oocytes and pre-implantation embryos are incubated in embryo culture medium pre-equilibrated for at least 4h in a CO_2 incubator (5% CO_2, 37°C, 95% humidity).

SEcuRe PROTOCOL APLICABILITY

The SEcuRe protocol can be used to perform **IVF with freshly harvested sperm** after applying the following modifications:
1. 2 males of the same line should be sacrificed and sperm of better quality (based on a visual assessment after 60 min preincubation) should be utilized in an IVF procedure. If sperm of both males show the same quality both can be used in an IVF. Sacrifice two males and dissect the cauda epididymides. Transfer the 2 cauda epididymides (after removal of fat and blood) of each male to separate dishes into the oil next to the c-TYH (or FERTIUP® PM) drop. After nicking the tissue drag sperm with watchmaker forceps into the drop.
2. Sperm should be allowed capacitation for 60 min in c-TYH (or FERTIUP® PM);
3. 0.25 mM GSH concentration should be used in the mRVF. For this purpose:

- Prepare a 100x $CaCl_2$ x $2H_2O$ stock solution (310 mM) by dissolving 0.4558 g of $CaCl_2$ x $2H_2O$ in 10 ml of embryo-grade water
- Filter the solution through a 0.22 µm filter and store aliquots at -20°C for a maximum of 6 months
- On the day of IVF thaw an aliquot of $CaCl_2$ at room temperature
- Add 150 µl of 100x $CaCl_2$ to 15 ml of RVF medium and mix gently
- Place 1 ml of RVF medium supplemented with $CaCl_2$ in a tube containing 30.7 mg of GSH and vortex
- Add 10 µl of this solution to 4 ml of RVF medium supplemented with $CaCl_2$, mix gently and filter using 0.22 µm syringe end filter

4. Oocytes from a maximum of 5 females should be placed in a 200 µl drop of mRVF;
5. 5 µl of sperm suspension should be added to the oocytes. If the removal of cumulus cells assessed after 20 min of incubation is poor, indicating insufficient motility or concentration of sperm, additional 5 µl of the sperm suspension should be transferred to the fertilization medium.

The SEcuRe protocol can also be used with **samples cryopreserved according to Ostermeier *et al.*** (2008) approach routinely used by The Jackson Laboratory. In this case, the entire sperm suspension should be expelled into the center of a 6-cm dish and 30 µl of that suspension should be added to a 90 µl drop of c-TYH (or FERTIUP PM®). Subsequently, IVF should be conducted according to the standard protocol described here (see Steps 23-28).

MATERIALS TEXT

L-Glutamine **Sigma Aldrich Catalog #G8540**

Citation: Magdalena Wigger, Simon E TrÃÂ¶der, Branko Zevnik ( ). A simple and economic protocol for efficient in vitro fertilization using cryopreserved mouse sperm. https://dx.doi.org/10.17504/protocols.io.bx2spqee

Water (for embryo transfer sterile-filtered BioXtra suitable for mouse embryo cell culture) **Sigma Aldrich Catalog #W1503**

Raffinose pentahydrate **Sigma Aldrich Catalog #R7630**

Skim Milk **Becton-Dickinson Catalog #232100**

FERTIUP Mouse Sperm Preincubation Medium: PM **Cosmo Bio Catalog #KYD-002-EX-X5**

Volumetric flasks Class B transparent 25 ml **Carl Roth Catalog #671941**

NaCl **Sigma Aldrich Catalog #S5886**

KCl **Sigma Aldrich Catalog #P5405**

MgSO4 x 7H20 **Sigma Aldrich Catalog #M2773**

KH2PO4 **Sigma Aldrich Catalog #P5655**

D-Glucose **Sigma Aldrich Catalog #G6152**

Penicillin G **Sigma Aldrich Catalog #P7794**

Streptomycin **Sigma Aldrich Catalog #S1277**

NaHCO3 **Sigma Aldrich Catalog #S8761**

Gibco™ Sodium Pyruvate (100mM) **Fisher Scientific Catalog #11-360-070**

Methyl-β-cyclodextrin **Sigma Aldrich Catalog #C4555**

Polyvinylalcohol (PVA) **Sigma Aldrich Catalog #P8136**

CaCl2 x 2H2O **Sigma Aldrich Catalog #C7902**

Cook Medical RVFE medium (IVF) (fertilization medium) **Cook Medical Catalog #K-RVFE-50**

L-Glutathione reduced **Sigma Aldrich Catalog #G4251**

MiniStraw 0.25 ml clear **Minitube Catalog #13407/0010**

Citation: Magdalena Wigger, Simon E TrÃÂ¶der, Branko Zevnik ( ). A simple and economic protocol for efficient in vitro fertilization using cryopreserved mouse sperm. https://dx.doi.org/10.17504/protocols.io.bx2spqee

Falcon® 35 mm TC-treated Easy-Grip Style Cell Culture Dish **Corning Catalog #353001**

Falcon® 60 mm TC-treated Easy-Grip Style Cell Culture Dish **Corning Catalog #353004**

Paraffin oil **Sigma Aldrich Catalog #76235**

Dulbecco's Phosphate Buffered Saline **Sigma Aldrich Catalog #D8537**

Sealing ball metal for 0.25 ml straws **Minitube Catalog #13400/9970**

Pipette tips Cell-Saver 200 µl farblos **Biozym Scientific GmbH Catalog #729055**

Acrodisc® Syringe Filters with Supor® Membrane Sterile - 0.2 µm 32 mm **Pall Catalog #4652**

M2 Medium **Sigma Aldrich Catalog #M7167**

EmbryoMax® KSOM Mouse Embryo Media **Merck Millipore Catalog #MR-106-D**

Sperm cryopreservation

1 Prepare 20 straws for 2 sacrificed males of the same line. Use of a single male is possible as well but the number of straws and volume of media used needs to be reduced by 50%

2 Mark the straws at 2.3 cm and 4.0 cm at the open end and label them at the other end (cotton plug)

3 Attach a 1 ml syringe to the labeled end of the straw and aspirate Research Vitro Fert (RVF) medium (Cook Medical; K-RVFE-50) until the meniscus reached the 4.0 cm mark

4 Aspirate a 2.3 cm air into the straw and store the assembly until required

5 Place a 120 µl drop of the gCPA (for preparation see Guidelines & Warnings) in a 35-mm culture dish, cover it with paraffin oil and add another 120 µl of gCPA into the drop to obtain a tall, semi-spherical 240 µl drop (for 4 cauda epididymis pooled from 2 males)

6 Sacrifice 2 males, collect the cauda epididymides and vasa deferentia in DPBS and clean them of fat and the testicular artery to avoid contaminating the semen with blood

7 Dry the cauda epididymides on a tissue (to avoid dilution of gCPA with DPBS), transfer to a 240 µl drop of prewarmed gCPA (on a 37°C hot plate for at least 5 min) and make 6-7 cuts across the cauda epididymis with a pair of micro spring

Citation: Magdalena Wigger, Simon E TrÃÂ¶der, Branko Zevnik ( ). A simple and economic protocol for efficient in vitro fertilization using cryopreserved mouse sperm. https://dx.doi.org/10.17504/protocols.io.bx2spqee

scissors

8 Place the dish on a 37°C hot plate for 3 min and gently swirl every min for 20 sec to help the sperm disperse from the tissue

9 Divide the sperm suspension into 20 aliquots of 10 µl on a 10-cm culture dish lid avoiding carryover of paraffin oil into the aliquots (clean the pipette tip from the outside with a tissue to remove the oil each time before placing a 10 µl aliquot on the dish lid)

10 Aspirate each 10 µl drop into a separate freezing straw followed by 2.3 cm air

11 Seal the straws (e.g. with a heat sealer or metal balls) and place them in liquid nitrogen gas phase for 10 min. Note: We use a custom-made metal inlay for this purpose but self-made or purchased freezing canisters can be used, too (e.g. http://card.medic.kumamoto-u.ac.jp/card/english/sigen/manual/spfreeze.html#canister or KYD-S018 from Cosmo Bio)

12 Transfer the straws to the liquid nitrogen tank for long-term storage

Oocyte isolation

13 Ideally, the entire IVF procedure (oocyte preincubation, sperm preincubation and fertilization) should be performed in an incubator (5% CO_2, 37°C) at 5% O_2 but atmospheric O_2 concentration have been shown to work well, too

14 Prepare a 35-cm culture dish with a 90 µl drop of mRVF (for preparation see Guidelines & Warnings) covered with oil (for oocytes from a maximum of 3 females) and equilibrate it for at least 20 min in an atmosphere of mixed gas (5% CO_2, 5% O_2, 37°C)

15 Collect oviducts from superovulated females 15 hours after the hCG injection and clean them in DPBS

16 Transfer the oviducts into the paraffin oil next to the 90 µl drop of mRVF

17 Release oocyte clutches into the oil by ripping the ampulla with forceps and drag them through the oil into the fertilization drop

18 Incubate oocytes for 50 min before adding the sperm suspension (at least 30 min and no longer than 60 min) in an atmosphere of mixed gas (5% CO_2, 5% O_2, 37°C)

Sperm thawing and capacitation

19 Prepare a 35-mm culture dish (for each IVF experiment) with 90 µl c-TYH drop (for preparation see Guidelines & Warnings) covered with paraffin oil and equilibrate it overnight in a CO_2 incubator (5% CO_2, 37°C, 95% humidity) or at

Citation: Magdalena Wigger, Simon E TrÃÂ¶der, Branko Zevnik ( ). A simple and economic protocol for efficient in vitro fertilization using cryopreserved mouse sperm. https://dx.doi.org/10.17504/protocols.io.bx2spqee

least 20 min in the morning of the IVF in an atmosphere of mixed gas (5% CO_2, 5% O_2, 37°C)

20 Remove the required straw(s) from long-term storage in liquid nitrogen on the day of IVF, place in a dewar with liquid nitrogen and then quickly transfer into a 37°C water bath for 10 min

21 Dry the straw(s) with a tissue and cut the sealed end and the labeled end of the straw below the cotton plug

22 Expel 10µl sperm suspension into the center of a 90 µl c-TYH drop using a 1 ml syringe

23 Preincubate for 30 min in an atmosphere of mixed gas (5% CO_2, 5% O_2, 37°C) before the IVF procedure to allow capacitation of the sperm

In vitro fertilization

24 Add 10 µl of the sperm suspension taken from the edge of the c-TYH drop to the oocyte clutches with the help of a 200 µl cell-saver tip and incubate for 4 hours (at least 3 hours and no longer than 5 hours) in an atmosphere of mixed gas (5% CO_2, 5% O_2, 37°C)

25 Add another 10 µl of the sperm suspension to the fertilization medium if the removal of cumulus cells assessed after 20 min of incubation is poor, indicating insufficient motility or concentration of sperm

Embryo culture and transfer

26 Wash embryos after the IVF procedure through 10 drops of preincubated embryo culture medium (e.g. M16 or KSOM) and incubate overnight in embryo culture medium in groups of 15-50 embryos per drop (a 30 µl drop of embryo culture medium covered with paraffin oil) in a CO_2 incubator (5% CO_2, 37°C, 95% humidity)

27 The day after insemination determine fertilization rates (a percentage of the total number of inseminated oocytes that developed to the 2-cell stage)

28 Transfer 2-cell embryos into the oviducts of pseudo-pregnant 0.5 dpc females

Citation: Magdalena Wigger, Simon E TrÃÂ¶der, Branko Zevnik ( ). A simple and economic protocol for efficient in vitro fertilization using cryopreserved mouse sperm. https://dx.doi.org/10.17504/protocols.io.bx2spqee
