# Supplementary figures and images for: A simple and economic protocol for efficient in vitro fertilization using cryopreserved mouse sperm
Source: PLoS One. 2021 Oct 28;16(10):e0259202. doi: 10.1371/journal.pone.0259202 (PMC8553151; doi:10.1371/journal.pone.0259202)

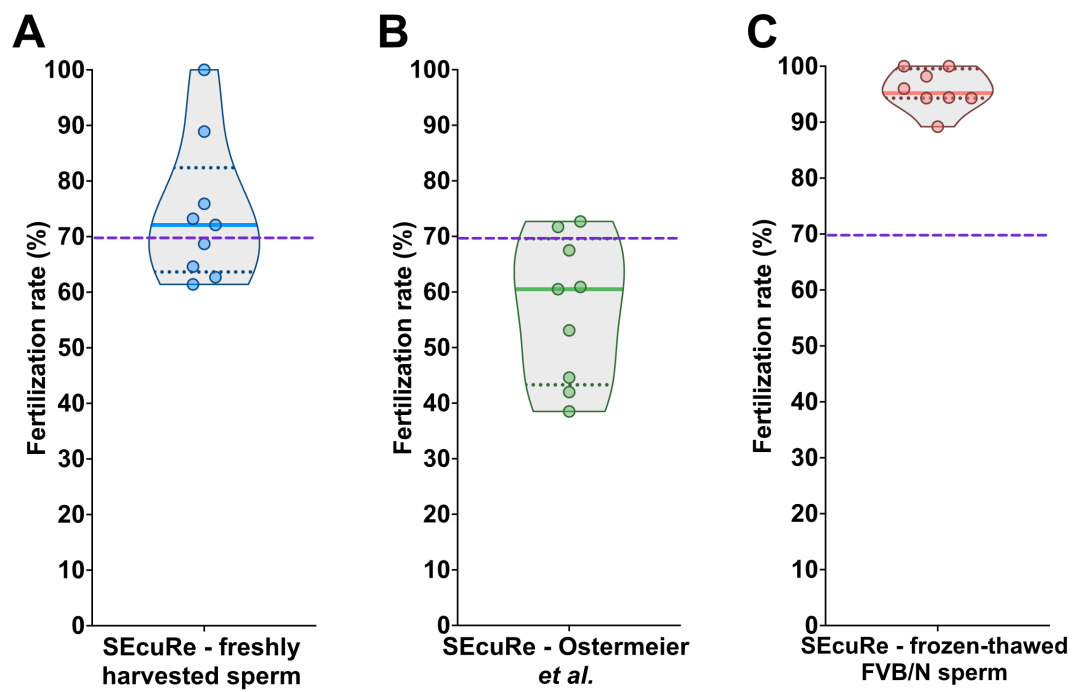

Supplement: S1 Fig — Fertilization rates achieved after IVF using the SEcuRe IVF protocol with sperm samples from different sources and genetic backgrounds. The SEcuRe approach can be employed for IVF procedures utilizing: (A) freshly harvested C57BL/6 sperm, (B) C57BL/6 sperm samples cryopreserved according to the Ostermeier et al. approach and (C) cryopreserved sperm samples from FVB/N background lines. Thick lines in the violin plots indicate median fertilization rates, dotted lines the first and the third quartile and points individual experiments (IVF procedures). For comparison, dashed lines demonstrate the median fertilization rate obtained with the SEcuRe protocol utilizing frozen-thawed C57BL/6 sperm in Fig 3A. (PDF) [file pone.0259202.s002.pdf]
