# Supplementary material for: A simple and economic protocol for efficient in vitro fertilization using cryopreserved mouse sperm
Source: PLoS One. 2021 Oct 28;16(10):e0259202. doi: 10.1371/journal.pone.0259202 (PMC8553151; doi:10.1371/journal.pone.0259202)
Supplement: S2 Table — (PDF) [file pone.0259202.s004.pdf]

S2 Table. Primary *in vitro* data (part 1) – SEcuRe protocol.

SEcuRe protocol							
**ID**	**No. of oocytes**	**No. of 2-cell embryos**	**Fertilization rate**	**ID**	**No. of oocytes**	**No. of 2-cell embryos**	**Fertilization rate**
1	104	44	42,3%	41	72	62	86,1%
2	31	20	64,5%	42	64	55	85,9%
3	95	56	58,9%	43	73	58	79,5%
4	103	93	90,3%	44	79	68	86,1%
5	104	68	65,4%	45	84	67	79,8%
6	37	36	97,3%	46	84	69	82,1%
7	30	21	70,0%	47	104	82	78,8%
8	79	64	81,0%	48	101	41	40,6%
9	60	51	85,0%	49	49	43	87,8%
10	58	43	74,1%	50	100	87	87,0%
11	47	30	63,8%	51	101	83	82,2%
12	129	77	59,7%	52	87	65	74,7%
13	83	67	80,7%	53	66	59	89,4%
14	97	79	81,4%	54	106	71	67,0%
15	81	57	70,4%	55	92	49	53,3%
16	131	105	80,2%	56	113	76	67,3%
17	92	78	84,8%	57	75	37	49,3%
18	68	64	94,1%	58	83	57	68,7%
19	59	52	88,1%	59	62	48	77,4%
20	104	100	96,2%	60	65	63	96,9%
21	87	69	79,3%	61	44	37	84,1%
22	85	70	82,4%	62	82	82	100,0%
23	118	89	75,4%	63	54	41	75,9%
24	112	102	91,1%	64	88	77	87,5%
25	62	57	91,9%	65	54	30	55,6%
26	66	65	98,5%	66	60	37	61,7%
27	94	57	60,6%	67	52	46	88,5%
28	108	103	95,4%	68	56	36	64,3%
29	76	53	69,7%	69	82	44	53,7%
30	34	27	79,4%	70	77	54	70,1%
31	71	46	64,8%	71	72	32	44,4%
32	76	55	72,4%	72	71	53	74,6%
33	44	38	86,4%	73	66	38	57,6%
34	76	71	93,4%	74	86	52	60,5%
35	59	55	93,2%	75	80	33	41,3%
36	74	67	90,5%	76	53	51	96,2%
37	54	41	75,9%	77	104	61	58,7%
38	48	42	87,5%	78	91	51	56,0%
39	45	34	75,6%	79	56	24	42,9%
40	63	57	90,5%	80	33	23	69,7%

S2 Table. Primary *in vitro* data (part 2) – SEcuRe protocol.

SEcuRe protocol							
ID	No. of oocytes	No. of 2-cell embryos	Fertilization rate	ID	No. of oocytes	No. of 2-cell embryos	Fertilization rate
81	67	61	91,0%	110	195	140	71,8%
82	57	27	47,4%	111	215	190	88,4%
83	69	54	78,3%	112	187	123	65,8%
84	71	68	95,8%	113	231	133	57,6%
85	70	39	55,7%	114	190	106	55,8%
86	106	69	65,1%	115	148	95	64,2%
87	41	29	70,7%	116	241	134	55,6%
88	120	53	44,2%	117	161	99	61,5%
89	390	310	79,5%	118	196	99	50,5%
90	83	63	75,9%	119	140	61	43,6%
91	55	45	81,8%	120	218	89	40,8%
92	68	37	54,4%	121	217	137	63,1%
93	112	41	36,6%	122	195	132	67,7%
94	70	17	24,3%	123	169	73	43,2%
95	90	30	33,3%	124	332	162	48,8%
96	108	28	25,9%	125	329	131	39,8%
97	88	35	39,8%	126	135	42	31,1%
98	85	26	30,6%	127	216	44	20,4%
99	100	37	37,0%	128	220	75	34,1%
100	121	38	31,4%	129	134	30	22,4%
101	60	14	23,3%	130	313	81	25,9%
102	331	266	80,4%	131	346	116	33,5%
103	342	271	79,2%	132	237	72	30,4%
104	418	258	61,7%	133	197	62	31,5%
105	349	255	73,1%	134	304	113	37,2%
106	385	243	63,1%	135	246	66	26,8%
107	377	155	41,1%	136	128	33	25,8%
108	258	218	84,5%	137	279	195	69,9%
109	198	93	47,0%				
