# Supplementary material for: A simple and economic protocol for efficient in vitro fertilization using cryopreserved mouse sperm
Source: PLoS One. 2021 Oct 28;16(10):e0259202. doi: 10.1371/journal.pone.0259202 (PMC8553151; doi:10.1371/journal.pone.0259202)
Supplement: S3 Table — (PDF) [file pone.0259202.s005.pdf]

S3 Table. Primary *in vitro* data – CARD Set protocol.

CARD Set protocol			
**ID**	**No. of oocytes**	**No. of 2-cell embryos**	**Fertilization rate**
**1**	342	233	68.1%
**2**	138	61	44.2%
**3**	339	139	41.0%
**4**	406	319	78.6%
**5**	441	343	77.8%
**6**	76	58	76.3%
**7**	401	131	32.7%
**8**	318	184	57.9%
**9**	121	83	68.6%
