# Supplementary material for: A simple and economic protocol for efficient in vitro fertilization using cryopreserved mouse sperm
Source: PLoS One. 2021 Oct 28;16(10):e0259202. doi: 10.1371/journal.pone.0259202 (PMC8553151; doi:10.1371/journal.pone.0259202)
Supplement: S4 Table — (PDF) [file pone.0259202.s006.pdf]

S4 Table. Primary *in vitro* data – Ostermeier *et al.* protocol.

Ostermeier *et al.* protocol							
**ID**	**No. of oocytes**	**No. of 2-cell embryos**	**Fertilization rate**	**ID**	**No. of oocytes**	**No. of 2-cell embryos**	**Fertilization rate**
**1**	88	36	40.9%	**41**	67	21	31.3%
**2**	48	26	54.2%	**42**	59	15	25.4%
**3**	49	23	46.9%	**43**	92	29	31.5%
**4**	61	28	45.9%	**44**	15	3	20.0%
**5**	36	21	58.3%	**45**	62	15	24.2%
**6**	32	17	53.1%	**46**	69	18	26.1%
**7**	35	25	71.4%	**47**	205	70	34.1%
**8**	29	18	62.1%	**48**	87	24	27.6%
**9**	135	87	64.4%	**49**	69	19	27.5%
**10**	101	67	66.3%	**50**	81	31	38.3%
**11**	40	19	47.5%	**51**	54	12	22.2%
**12**	68	38	55.9%	**52**	35	26	74.3%
**13**	76	61	80.3%	**53**	94	37	39.4%
**14**	139	75	54.0%	**54**	66	20	30.3%
**15**	86	66	76.7%	**55**	90	36	40.0%
**16**	87	48	55.2%	**56**	127	80	63.0%
**17**	34	14	41.2%	**57**	62	21	33.9%
**18**	40	16	40.0%	**58**	99	27	27.3%
**19**	34	16	47.1%	**59**	52	19	36.5%
**20**	71	27	38.0%	**60**	95	58	61.1%
**21**	60	12	20.0%	**61**	58	17	29.3%
**22**	135	48	35.6%	**62**	76	30	39.5%
**23**	58	13	22.4%	**63**	54	15	27.8%
**24**	100	30	30.0%	**64**	113	44	38.9%
**25**	40	9	22.5%	**65**	87	24	27.6%
**26**	29	9	31.0%	**66**	139	55	39.6%
**27**	68	20	29.4%	**67**	63	24	38.1%
**28**	27	7	25.9%	**68**	379	122	32.2%
**29**	48	18	37.5%	**69**	470	225	47.9%
**30**	53	15	28.3%	**70**	220	76	34.5%
**31**	45	12	26.7%	**71**	65	52	80.0%
**32**	31	12	38.7%	**72**	375	192	51.2%
**33**	31	12	38.7%	**73**	51	30	58.8%
**34**	47	18	38.3%	**74**	40	22	55.0%
**35**	57	19	33.3%	**75**	39	17	43.6%
**36**	28	9	32.1%	**76**	248	125	50.4%
**37**	29	11	37.9%	**77**	454	112	24.7%
**38**	34	7	20.6%	**78**	113	55	48.7%
**39**	73	26	35.6%	**79**	371	142	38.3%
**40**	92	34	37.0%				
