# Supplementary material for: A simple and economic protocol for efficient in vitro fertilization using cryopreserved mouse sperm
Source: PLoS One. 2021 Oct 28;16(10):e0259202. doi: 10.1371/journal.pone.0259202 (PMC8553151; doi:10.1371/journal.pone.0259202)
Supplement: S5 Table — (PDF) [file pone.0259202.s007.pdf]

S5 Table. Primary data – FERTIUP® PM – c-TYH comparison.

ID	FERTIUP® PM					c-TYH				
	No. of oocytes	No. of 2-cell embryos	Fertilization rate	No. of blastocysts	Blastocyst formation rate	No. of oocytes	No. of 2-cell embryos	Fertilization rate	No. of blastocysts	Blastocyst formation rate
1	147	115	78,23%	102	88,70%	108	89	82,41%	78	87,64%
2	98	76	77,55%	59	77,63%	108	86	79,63%	54	62,79%
3	52	44	84,62%	36	81,82%	67	55	82,09%	42	76,36%
