# Supplementary material for: A simple and economic protocol for efficient in vitro fertilization using cryopreserved mouse sperm
Source: PLoS One. 2021 Oct 28;16(10):e0259202. doi: 10.1371/journal.pone.0259202 (PMC8553151; doi:10.1371/journal.pone.0259202)
Supplement: S6 Table — (PDF) [file pone.0259202.s008.pdf]

**S6 Table. Primary *in vivo* data – SEcuRe protocol.**

| SEcuRe protocol | | | | |
|---|---|---|---|---|
| **ID** | **No. of embryos transferred** | **No. of recipient mice** | **No. of born pups** | **Birth rate** |
| **1** | 88 | 4 | 27 | 30,7% |
| **2** | 88 | 4 | 28 | 31,8% |
| **3** | 82 | 4 | 44 | 53,7% |
| **4** | 82 | 4 | 35 | 42,7% |
| **5** | 110 | 5 | 33 | 30,0% |
| **6** | 150 | 6 | 36 | 24,0% |
| **7** | 132 | 6 | 36 | 27,3% |
| **8** | 69 | 3 | 26 | 37,7% |
| **9** | 88 | 4 | 19 | 21,6% |
| **10** | 88 | 4 | 27 | 30,7% |
| **11** | 88 | 4 | 29 | 33,0% |
| **12** | 44 | 2 | 18 | 40,9% |
| **13** | 44 | 2 | 19 | 43,2% |
| **14** | 88 | 4 | 25 | 28,4% |
| **15** | 77 | 4 | 22 | 28,6% |
| **16** | 60 | 3 | 19 | 31,7% |
| **17** | 66 | 3 | 14 | 21,2% |
| **18** | 88 | 4 | 38 | 43,2% |
| **19** | 88 | 4 | 16 | 18,2% |
| **20** | 21 | 1 | 7 | 33,3% |
| **21** | 110 | 5 | 43 | 39,1% |
| **22** | 198 | 9 | 53 | 26,8% |
| **23** | 105 | 5 | 29 | 27,6% |
| **24** | 42 | 2 | 13 | 31,0% |
| **25** | 22 | 1 | 5 | 22,7% |
| **26** | 44 | 2 | 15 | 34,1% |
| **27** | 30 | 2 | 5 | 16,7% |
| **28** | 61 | 3 | 19 | 31,1% |
| **29** | 110 | 5 | 30 | 27,3% |
| **30** | 66 | 3 | 20 | 30,3% |
| **31** | 66 | 3 | 21 | 31,8% |
| **32** | 44 | 2 | 5 | 11,4% |
| **33** | 16 | 1 | 9 | 56,3% |
