# Supplementary material for: A simple and economic protocol for efficient in vitro fertilization using cryopreserved mouse sperm
Source: PLoS One. 2021 Oct 28;16(10):e0259202. doi: 10.1371/journal.pone.0259202 (PMC8553151; doi:10.1371/journal.pone.0259202)
Supplement: S7 Table — (PDF) [file pone.0259202.s009.pdf]

S7 Table. Primary *in vivo* data – CARD Set protocol.

CARD Set protocol				
**ID**	**No. of embryos transferred**	**No. of recipient mice**	**No. of born pups**	**Birth rate**
**1**	56	3	10	17,9%
**2**	20	1	4	20,0%
**3**	66	3	25	37,9%
**4**	82	4	25	30,5%
**5**	24	1	11	45,8%
**6**	44	2	21	47,7%
**7**	22	1	8	36,4%
**8**	88	4	19	21,6%
**9**	35	2	13	37,1%
