# Supplementary material for: A simple and economic protocol for efficient in vitro fertilization using cryopreserved mouse sperm
Source: PLoS One. 2021 Oct 28;16(10):e0259202. doi: 10.1371/journal.pone.0259202 (PMC8553151; doi:10.1371/journal.pone.0259202)
Supplement: S8 Table — (PDF) [file pone.0259202.s010.pdf]

S8 Table. Primary *in vivo* data – Ostermeier *et al.* protocol.

Ostermeier *et al.* protocol				
**ID**	**No. of embryos transferred**	**No. of recipient mice**	**No. of born pups**	**Birth rate**
**1**	16	1	5	31,3%
**2**	54	3	6	11,1%
**3**	39	2	12	30,8%
**4**	88	4	25	28,4%
**5**	28	2	12	42,9%
**6**	56	3	15	26,8%
**7**	44	2	15	34,1%
**8**	44	2	15	34,1%
**9**	45	2	18	40,0%
**10**	12	1	2	16,7%
**11**	29	2	13	44,8%
**12**	75	3	17	22,7%
**13**	52	2	11	21,2%
**14**	20	1	3	15,0%
**15**	95	5	26	27,4%
**16**	36	2	14	38,9%
