# Supplementary material for: A simple and economic protocol for efficient in vitro fertilization using cryopreserved mouse sperm
Source: PLoS One. 2021 Oct 28;16(10):e0259202. doi: 10.1371/journal.pone.0259202 (PMC8553151; doi:10.1371/journal.pone.0259202)
Supplement: S9 Table — (PDF) [file pone.0259202.s011.pdf]

S9 Table. Number of mice used during IVF procedures and embryo transfers.

Protocol	Sacrificed			Recipients used
	females	males	total	
**SEcuRe**	661	268	929	118
**CARD Set**	129	15	144	20
**Ostermeier *et al.***	350	78	428	37
		**Total**	1501	175
